# Impact of intensive care unit admission during handover on mortality: propensity matched cohort study

**DOI:** 10.31744/einstein_journal/2021AO5748

**Published:** 2021-06-10

**Authors:** Thais Dias Midega, Newton Carlos Viana Leite, Antonio Paulo Nassar, Roger Monteiro Alencar, Antonio Capone, Leonardo José Rolim Ferraz, Thiago Domingos Corrêa

**Affiliations:** 1 Hospital Israelita Albert Einstein São PauloSP Brazil Hospital Israelita Albert Einstein, São Paulo, SP, Brazil.; 2 Hospital Israelita Albert Einstein Hospital Municipal Dr. Moysés Deutsch São PauloSP Brazil Hospital Municipal Dr. Moysés Deutsch; Hospital Israelita Albert Einstein, São Paulo, SP, Brazil.

**Keywords:** Patient handoff, Patient safety, Patient outcome assessment, Intensive care units/statistics & numerical data, Communication, Patient readmission, Patient discharge, Hospital mortality, Health resources/statistics & numerical data

## Abstract

**Objective:**

To investigate the impact of intensive care unit admission during medical handover on mortality.

**Methods:**

*Post-hoc* analysis of data extracted from a prior study aimed at addressing the impacts of intensive care unit readmission on clinical outcomes. This retrospective, single-center, propensity-matched cohort study was conducted in a 41-bed general open-model intensive care unit. Patients were assigned to one of two cohorts according to time of intensive care unit admission: Handover Group (intensive care unit admission between 6:30 am and 7:30 am or 6:30 pm and 7:30 pm) or Control Group (intensive care unit admission between 7:31 am and 6:29 pm or 7:31 pm and 6:29 am). Patients in the Handover Group were propensity-matched to patients in the Control Group at a 1:2 ratio.

**Results:**

A total of 6,650 adult patients were admitted to the intensive care unit between June 1^st^ 2013 and May 31^st^ 2015. Following exclusion of non-eligible participants, 5,779 patients (389; 6.7% and 5,390; 93.3%, Handover and Control Group) were deemed eligible for propensity score matching. Of these, 1,166 were successfully matched (389; 33.4% and 777; 66.6%, Handover and Control Group). Following propensity-score matching, intensive care unit admission during handover was not associated with increased risk of intensive care unit (OR: 1.40; 95%CI: 0.92-2.11; p=0.113) or in-hospital (OR: 1.23; 95%CI: 0.85-1.75; p=0.265) mortality.

**Conclusion:**

Intensive care unit admission during medical handover did not affect in-hospital mortality in this propensity-matched, single-center cohort study.

## INTRODUCTION

Rising demand for intensive care unit (ICU) beds worldwide reflects increasing life expectancy and growing prevalence of chronic conditions.^(^^1^^)^ Given the limited availability of ICU beds, improvement of ICU organizational and operational characteristics is vital for enhanced efficiency and better outcomes. In Brazil, most ICUs have full time, *i.e.*, 24 hours a day, 7 days a week in-house intensivist coverage.^(^^2^^)^ Therefore, frequent transitions of care between health care professionals are expected.

Handover is the transfer of patient information, therapeutic plans and responsibilities from a departing to an oncoming provider.^(^^3^^)^ Associations between inadequate handover and *(poorer?)* clinical outcomes have been reported.^(^^4^^–^^6^^)^ For instance, in a study conducted at the emergency department, inadequate handover had adverse effects on approximately 5% of patients, resulting in delayed therapy.^(^^7^^)^ A review of emergency department malpractice claims revealed a 24% rate of missed diagnosis due to inadequate handover.^(^^8^^)^ In another study, intraoperative anesthesia handover translated into a 43% higher chance of in-hospital mortality among patients submitted to cardiac surgery.^(^^9^^)^

The handover process is particularly challenging in ICU settings, given the complexity of critically ill patients and their propensity to sudden changes in clinical status.^(^^10^^)^ Patients admitted to ICU also tend to be unstable and may require timely resuscitative maneuvers, invasive procedures and therapeutic interventions within the first hours of admission.^(^^11^^)^ As a result, ICU admission during handover, when intensivists are diverted away from direct patient care, is likely to be associated with increased incidence of medical errors and unexpected adverse events.^(^^12^^)^

## OBJECTIVE

To examine the impact of critically ill patient admission to the intensive care unit during medical handover on in-hospital mortality at a tertiary care hospital, and to compare resource use and clinical outcomes between patients admitted to the intensive care unit during handover and patients admitted at different times.

## METHODS

### Study design and settings

This study is a *post-hoc* analysis of data extracted from a prior retrospective, single-center cohort study investigating the impacts of ICU readmission on resource use and clinical outcomes.^(^^13^^)^ The original study and this *post-hoc* analysis were approved by the Ethics Committee of *Hospital Israelita Albert Einstein* (HIAE) with waiver of informed consent (CAAE: 54065716.3.0000.0071; protocol: 1.464.901).

### Settings

This study was conducted at a private tertiary care hospital in São Paulo (SP), Brazil. The hospital in question had 662 inpatient beds, one general adult open-model ICU with 41 beds and 91 stepdown unit beds.

### Patients

Consecutive patients aged ≥18 years admitted to the ICU between June 1^st^ 2013 and May 31^st^ 2015 were included in the study. Patients with missing core data (age, sex, time of ICU admission, ICU admission diagnosis, Simplified Acute Physiology Score – SAPS – III upon ICU admission, ICU and hospital length of stay and vital status upon hospital discharge) were excluded.

### Data collection and study variables

Data were retrieved from the Epimed Monitor System^®^ (Epimed Solutions, Rio de Janeiro, RJ, Brazil). This system consists of an electronic structured case report form in which patient data are prospectively entered by trained ICU case managers.^(^^14^^)^ Variables collected included demographic characteristics, comorbidities, location prior to ICU admission, time of ICU admission and discharge, reason for ICU admission, SAPS III score upon ICU admission,^(^^15^^)^ ICU admission diagnosis, need of invasive support (vasopressors, mechanical ventilation, non-invasive mechanical ventilation – NIV – or renal replacement therapy – RRT) upon ICU admission and during ICU stay, destination after ICU discharge, ICU and hospital length of stay, frequency of ICU readmission, in-hospital mortality, mortality upon ICU discharge and 90-day mortality.

### Definitions

Handover was defined as the transfer of care from a departing to an oncoming intensivist. Handover time was defined as the following time periods: 6:30 am to 7:30 am and 6:30 pm to 7:30 pm. Patients were assigned to one of two cohorts according to time of ICU admission: Handover Group (ICU admission between 6:30 am and 7:30 am or 6:30 pm and 7:30 pm) or Control Group (admission between 7:31 am and 6:29 pm or 7:31 pm and 6:29 am).

### Intensive care unit characteristics and handover process

Intensive care unit physicians were on-site 24 hours a day at a 1:10 intensivist-to ICU bed ratio. There was no reduction in personnel or ICU activities during night shifts or weekends. Multidisciplinary clinical rounds involving ICU physicians, nurses, respiratory therapists, nutritionists, psychologists and clinical pharmacists were held daily. Intensive care unit admission decisions were made by intensivists on duty, whereas discharge was a consensus decision-making process involving intensivists on duty and primary care physicians, *i.e.*, physicians who will be in charge of patients outside the ICU.^(^^13^^)^ On weekdays, intensivists on duty were not always the same, since intensivists work 12-hour shifts.

Handover took place twice daily, at 7:00 am and 7:00 pm, when the departing intensivist shift end. Departing ICU physicians usually begin to prepare for handover 30 minutes ahead of handover time. The handover process usually lasted 30 minutes. There were no specific protocols or standardized tools to guide handover during the experimental period.

Handover from departing to oncoming ICU nurses and respiratory therapists took place at the same time as handover between intensivists.

### Statistical analysis

Categorical variables were expressed as absolute and relative frequencies. Continuous variables were expressed as medians and interquartile ranges (IQR). Normality was assessed using the Kolmogorov-Smirnov test. The Handover and the Control Group were compared. Categorical variables were compared using the χ^2^ or the Fisher’s exact test as appropriate. Continuous variables were compared using the independent *t* or the Mann-Whitney U test for non-normally distributed data.

Propensity-score matching was used to account for differences in patient characteristics so as to mitigate the effects of confounding. Propensity scores were estimated for each patient in the Handover Group using logistic regression conducted with 17 relevant characteristics (age, sex, SAPS III score, reason for ICU admission, admission source, systemic hypertension, *diabetes mellitus*, cancer, congestive heart failure, chronic obstructive pulmonary disease, chronic kidney disease, chronic kidney disease requiring long-term dialysis, liver cirrhosis and use of vasopressors, RRT, noninvasive mechanical ventilation or mechanical ventilation). Patients with missing data were excluded. A propensity score-matched cohort was constructed based on propensity score weighted estimators. Matching pairs were obtained using nearest neighbor matching without replacement, in which each patient in Handover Group was matched to two patients in the Control Group. A caliper width of 0.10 of the standard deviation of the logit of the propensity score was used for matching development.^(^^16^^,^^17^^)^

Statistical tests were 2-sided, and p values below 0.05 were considered statistically significant. No adjustments for multiplicity were made in the analysis. Statistical analyses were performed using IBM SPSS, version 22.0. Plots were generated using GraphPad Prism for Windows, version 6.00 (GraphPad software, California, USA).

## RESULTS

### Study population characteristics

**Figure 1:**
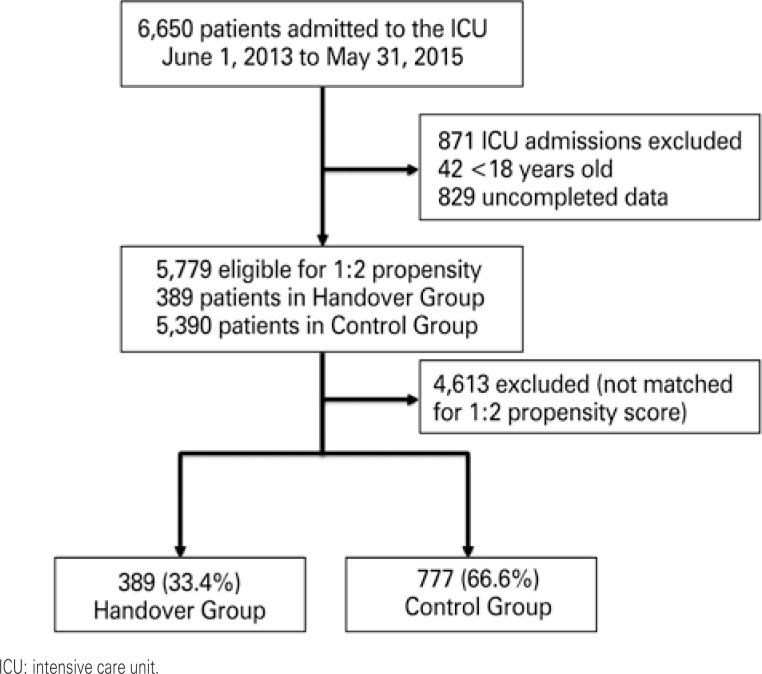
Patient flowchart

**Figure 2:**
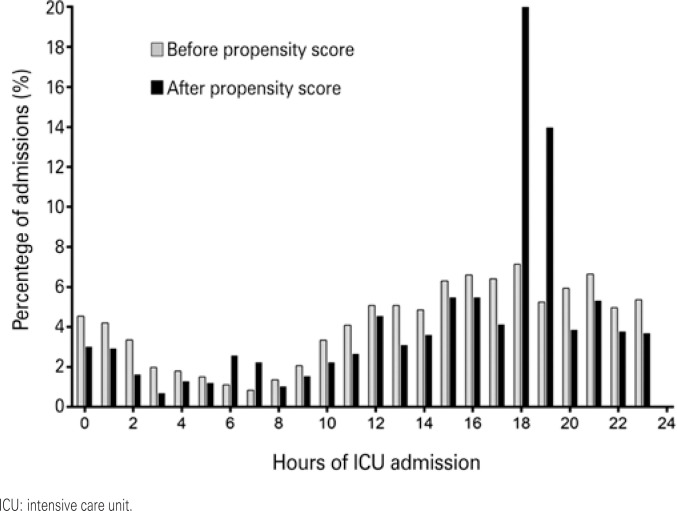
Percentage of intensive care unit admission per hour of the day

A total of 6,650 patients were admitted to ICU between June 2013 and May 2015. Of these, 871 patients admitted to ICU were excluded due to incomplete core data and/or age under eighteen years. The final sample comprised 5,779 patients (389; 6.7% in the Handover and 5,390; 93.3% in the Control Group). The median age of patients in this cohort was 67 (IQR of 53 to 80) years and 56.5% of patients were males. Out of 5,779 patients eligible for propensity score matching, 1,166 were successfully matched (389; 33.4% in the Handover and 777; 66.6% in the Control Group) (Figure 1). Time distribution of ICU admission across the study population (n=5,779 patients) is shown in the histogram in figure 2.

### Cohort prior to propensity score matching

Age, sex, SAPS III score, reason for ICU admission, admission source, admission diagnosis, frequency of comorbidities and need of support upon ICU admission, such as mechanical ventilation, NIV, RRT and vasopressors, did not differ between patients in the Handover and the Control Group prior to propensity score matching (Table 1).

**Table 1:** Characteristics of study participants prior to propensity score matching

Characteristics		All patients 5,779 (100.0%)	Handover Group 389 (6.7%)	Control Group 5,390 (93.3%)	p value**
Age, years		67 (53-80)	67 (52-80)	66 (53-80)	0.483#
Male		3,268 (56.5)	214 (55)	3,054 (56.7)	0.527&
SAPS III score§		43 (33-55)	43 (32-54)	43 (33-55)	0.537‡
Reason for ICU admission					0.563&
	Medical	3,423 (59.2)	225 (57.8)	3,198 (59.3)	
	Surgical	2,356 (40.8)	164 (42.2)	2,192 (40.7)	
Admission source					0.741&
	Operating room/procedure unit	2,313 (40.0)	155 (39.8)	2,158 (40)	
	Emergency department	2,217 (38.4)	148 (38.0)	2,069 (38.4)	
	Inpatient unit	557 (9.6)	35 (9.0)	522 (9.7)	
	Stepdown unit	377 (6.5)	24 (6.2)	353 (6.5)	
	Other*	315 (5.5)	27 (6.9)	288 (5.3)	
Underlying disease					
	Hypertension	3,132 (54.2)	210 (54.0)	2,922 (54.2)	0.925&
	*Diabetes mellitus*	1,838 (31.8)	124 (31.9)	1,714 (31.8)	0.979&
	Cancer	1,270 (22.0)	97 (24.9)	1,173 (21.8)	0.145&
Congestive heart failure		702 (12.2)	47 (12.1)	655 (12.2)	0.719&
	COPD	519 (9.0)	36 (9.3)	483 (9.0)	0.854&
	Chronic kidney disease requiring long-term dialysis	470 (8.1)	25 (6.4)	445 (8.3)	0.202&
	Chronic kidney disease	397 (6.9)	25 (6.4)	372 (6.9)	0.719&
	Liver cirrhosis	279 (4.8)	21 (5.4)	258 (4.8)	0.588&
Non-operative admission diagnosis					0.849&
	Sepsis	1,657 (48.4)	113 (50.2)	1,544 (48.3)	
	Cardiovascular	508 (14.8)	38 (16.9)	470 (14.7)	
	Neurologic	384 (11.2)	22 (9.8)	362 (11.3)	
	Respiratory	326 (9.5)	22 (9.8)	304 (9.5)	
	Gastrointestinal	238 (6.7)	17 (7.6)	221 (6.9)	
	Metabolic	98 (2.9)	3 (1.3)	95 (3.0)	
	Trauma	94 (2.7)	4 (1.8)	90 (2.8)	
	Other medical conditions	69 (2.0)	3 (1.3)	66 (2.1)	
	Renal diseases	30 (0.9)	2 (0.9)	28 (0.9)	
	Hematologic	19 (0.6)	1 (0.4)	18 (0.6)	
Operative admission diagnosis					0.748&
	Cardiovascular	643 (27.3)	42 (25.6)	601 (27.4)	
	Gastrointestinal	545 (23.1)	34 (20.7)	511 (23.3)	
	Orthopedic	394 (16.7)	26 (15.9)	368 (16.8)	
	Renal	286 (12.1)	25 (15.2)	261 (11.9)	
	Neurologic	235 (10.0)	15 (9.1)	220 (10.0)	
	Respiratory	203 (8.6)	19 (11.6)	184 (8.4)	
	Gynecologic	47 (2.0)	3 (1.8)	44 (2.0)	
	Trauma	3 (0.1)	0 (0.0)	3 (0.1)	
Support at ICU admission					
	Mechanical ventilation	953 (16.5)	57 (14.7)	896 (16.6)	0.312&
	Non-invasive ventilation	521 (9.0)	41 (10.5)	480 (8.9)	0.277&
	Vasopressors	852 (14.7)	48 (12.3)	804 (14.9)	0.166&
	RRT	24 (0.4)	0 (0.0)	24 (0.4)	0.187&
Destination upon ICU discharge					0.338&
	Stepdown unit	3,152 (54.5)	216 (55.5)	2,936 (54.5)	
	Inpatient unit	2,057 (35.6)	134 (34.4)	1,923 (35.7)	
	Other/unknown*	237 (4.1)	11 (2.8)	226 (4.2)	

Results expressed as median (interquartile range) or n (%).

** p values were calculated as follows:

# independent *t* test;

& χ^2^ test or

‡ Mann-Whitney U test;

§ SAPS III scores ranging from 0 to 217, with higher scores indicating more severe illness and higher risk of death;

* other hospital or home care.

SAPS: Simplified Acute Physiology Score; ICU: intensive care unit; COPD: chronic obstructive pulmonary disease; RRT: renal replacement therapy.

In-hospital mortality was 14.1% (55/389 patients) and 11.7% (628/5,390 patients) in the Handover and the Control Groups, respectively (odds ratio – OR: 1.25; 95% confidence interval – 95%CI: 0.92-1.68; p=0.142). Resource use, expressed as need of vasopressors, mechanical ventilation, NIV or RRT, did not differ between groups. Length of ICU, length of hospital stay and frequency of ICU readmissions were also similar between groups (Table 2).

**Table 2:** Outcomes prior to propensity score matching

Characteristics		All patients 5,779 (100.0%)	Handover Group 389 (6.7%)	Control Group 5,390 (93.3%)	p value*
ICU mortality		479 (8.3)	42 (10.8)	437 (8.1)	0.063†
90-day mortality		639 (11.1)	51 (13.1)	588 (10.9)	0.181†
In-hospital mortality		683 (11.8)	55 (14.1)	628 (11.7)	0.142†
Support during ICU stay					
	Vasopressors	1,586 (27.4)	98 (25.2)	1,488 (27.6)	0.303†
	Mechanical ventilation	1,401 (24.2)	88 (22.6)	1,313 (24.4)	0.440†
	Non-invasive ventilation	1,438 (24.9)	101 (26.0)	1,337 (24.8)	0.610†
	RRT	566 (9.8)	30 (7.7)	536 (9.9)	0.153†
ICU length of stay, days		2 (1-3)	2 (1-3)	2 (1-3)	0.117‡
Hospital length of stay, days		9 (5-20)	9 (9-18)	9 (5-20)	0.673‡
ICU readmission		576 (10.0)	38 (9.8)	538 (10.0)	0.891†

Results expressed as median (interquartile range) or n (%).

* p values were calculated using:

† the χ^2^ test or

‡ the Mann-Whitney U test.

ICU: intensive care unit; RRT: renal replacement therapy.

### Cohort following propensity score matching

The propensity-matched cohort had a median age of 67 (IQR of 53 to 80) years; 54.9% (640/1,166) of patients were males with median SAPS III of 43 (IQR of 32 to 55) (Table 3). Groups in this study were well balanced with respect to age, sex, SAPS III upon ICU admission, reason for ICU admission, admission source, prevalence of comorbidities, ICU admission diagnosis and need of supportive therapy upon index ICU admission (Table 3).

**Tabel 3:** Characteristics of study participants following propensity score matching

Characteristics		All patients 1,166 (100.0%)	Handover Group 389 (33.4%)	Control Group 777 (66.6%)	p value**
Age, years		67 (53-80)	67 (52-80)	68 (53-80)	0.880#
Male		640 (54.9)	214 (55)	426 (54.8)	0.952&
SAPS III^§^		43 (32-55)	43 (32-54)	43 (33-55)	0.621‡
Reason for ICU admission					0.750&
	Medical	682 (58.5)	225 (57.8)	457 (58.8)	
	Surgical	484 (41.5)	164 (42.2)	320 (41.2)	
Admission source					0.863&
	Operating room/procedure unit	464 (39.8)	155 (39.8)	309 (39.8)	
	Emergency department	454 (38.9)	148 (38.0)	306 (39.4)	
	Inpatient unit	91 (7.8)	35 (9.0)	56 (7.2)	
	Stepdown unit	76 (6.5)	24 (6.2)	52 (6.7)	
	Other*	81 (6.9)	27 (6.9)	54 (6.9)	
Underlying disease					
	Hypertension	620 (53.2)	210 (54.0)	410 (52.8)	0.694&
	*Diabetes mellitus*	391 (33.5)	124 (31.9)	267 (34.4)	0.396&
	Cancer	305 (26.2)	97 (24.9)	208 (26.8)	0.502&
	Congestive heart failure	152 (13.0)	47 (12.1)	105 (13.5)	0.494&
	COPD	111 (9.5)	36 (9.3)	75 (9.7)	0.827&
	Chronic kidney disease requiring long-term dialysis	85 (7.3)	25 (6.4)	60 (7.7)	0.422&
	Chronic kidney disease	76 (6.5)	25 (6.4)	51 (6.6)	0.929&
	Liver cirrhosis	61 (5.2)	21 (5.4)	40 (5.1)	0.856&
Non-operative admission diagnosis					0.579&
	Sepsis	332 (48.7)	113 (50.2)	219 (47.9)	
	Cardiovascular	105 (15.4)	38 (16.9)	67 (14.7)	
	Neurologic	86 (12.6)	22 (9.8)	64 (14.0)	
	Respiratory	64 (9.4)	22 (9.8)	42 (9.2)	
	Gastrointestinal	44 (6.5)	17 (7.6)	27 (5.9)	
	Metabolic	17 (2.5)	3 (1.3)	14 (3.1)	
	Trauma	15 (2.2)	4 (1.8)	11 (2.4)	
	Other medical conditions	12 (1.8)	3 (1.3)	9 (2.0)	
	Renal diseases	3 (0.4)	2 (0.9)	1 (0.2)	
	Hematologic	4 (0.6)	1 (0.4)	3 (0.7)	
Operative admission diagnosis					0.876&
	Cardiovascular	116 (24.0)	42 (25.6)	74 (23.1)	
	Gastrointestinal	102 (21.1)	34 (20.7)	68 (21.3)	
	Orthopedic	76 (15.7)	26 (15.9)	50 (15.6)	
	Renal	70 (14.5)	25 (15.2)	45 (14.1)	
	Neurologic	54 (11.2)	15 (9.1)	39 (12.2)	
	Respiratory	52 (10.7)	19 (11.6)	33 (10.3)	
	Gynecologic	14 (2.9)	3 (1.8)	11 (3.4)	
Support upon ICU admission					
	Mechanical ventilation	185 (15.9)	57 (14.7)	128 (16.5)	0.422&
	Non-invasive ventilation	113 (9.7)	41 (10.5)	72 (9.3)	0.488&
	Vasopressors	168 (14.4)	48 (12.3)	120 (15.4)	0.155&
	RRT	2 (0.2)	0 (0.0)	2 (0.3)	0.555&
Destination upon ICU discharge					0.723&
	Stepdown unit	645 (55.3)	216 (55.5)	429 (55.2)	
	Inpatient unit	407 (34.9)	134 (34.4)	273 (35.1)	
	Other/unknown*	40 (3.4)	11 (2.8)	29 (3.7)	

Results expressed as median (interquartile range) or n (%).

** p values were calculated using:

# the independent *t* test;

& the χ^2^ test or

‡ the Mann-Whitney U test;

* other hospital or home care.

SAPS: Simplified Acute Physiology Score; ICU: intensive care unit; COPD: chronic obstructive pulmonary disease; RRT: renal replacement therapy.

Intensive care unit mortality was 10.8% (42/389 patients) and 8.0% (62/777 patients) in the Handover and the Control Groups, respectively (OR: 1.40; 95%CI: 0.92-2.11; p=0.113). In-hospital mortality was 14.1% (55/389 patients) and 11.8% (92/777 patients) in the Handover and the Control Groups, respectively (OR: 1.23; 95%CI: 0.85-1.75; p=0.265). Need of vasopressors, mechanical ventilation, NIV or RRT, ICU and hospital length of stay and frequency of ICU readmissions did not differ between groups (Table 4).

**Tabel 4:** Outcomes after propensity score matching

Characteristics		All patients 1,166 (100.0%)	Handover Group 389 (33.4%)	Control Group 777 (66.6%)	p value*
ICU mortality		104 (8.9)	42 (10.8)	62 (8.0)	0.111†
90-day mortality		139 (11.9)	51 (13.1)	88 (11.3)	0.375†
In-hospital mortality		147 (12.6)	55 (14.1)	92 (11.8)	0.265†
Support during ICU stay					
	Vasopressors	302 (25.9)	98 (25.2)	204 (26.3)	0.696†
	Mechanical ventilation	270 (23.2)	88 (22.6)	182 (23.4)	0.760†
	Non-invasive ventilation	321 (27.5)	101 (26.0)	220 (28.3)	0.397†
	Renal replacement therapy	94 (8.1)	30 (7.7)	64 (8.2)	0.756†
ICU length of stay, days		2 (1-4)	2 (1-3)	2 (1-4)	0.258‡
Hospital length of stay, days		10 (5-20)	9 (5-18)	10 (5-20)	0.689‡
ICU readmission		120 (10.3)	38 (9.8)	82 (10.6)	0.678†

Results expressed as median (interquartile range) or n (%).

* p values were calculated using:

† the χ^2^ test or

‡ the Mann-Whitney U test.

ICU: intensive care unit.

## DISCUSSION

The main finding of this retrospective, single-center, propensity-matched cohort study was that ICU admission during medical handover did not affect resource use or clinical outcomes.

The concept that patient admission during handover might impact urgent patient care and clinical outcomes has been recently addressed in a retrospective, single-center cohort study with septic patients presenting to the emergency department during nursing handover.^(^^3^^)^ That study failed to reveal significant differences in time to antibiotic administration, time to serum lactate result, time to obtain blood culture and in-hospital mortality between patients who arrived at the emergency department during nursing handover relative to those who did not arrive during handover time.^(^^3^^)^

To the best of our knowledge, the impact of intensive care unit admission during handover on clinical outcomes has not been addressed so far. However, there is some evidence that patient care during handover is associated with worse outcomes in surgical patients. A retrospective, population-based cohort study including 313,066 patients submitted to major surgery in Canada compared the impact of complete handover of intraoperative anesthesia care between anesthesiologists with no handover of anesthesia care on clinical outcomes.^(^^18^^)^ In that study, patients experiencing transition of medical care during surgery had a higher risk of adverse postoperative outcomes.^(^^18^^)^ In another retrospective, single-center study, handover of anesthesia care during cardiac surgery was associated with a 43% higher risk of in-hospital mortality.^(^^9^^)^ Likewise, patients who experience transition of anesthesia care during surgery have worse outcomes relative to those who do not, mostly due to missed information.^(^^18^^)^

The impact of communication errors during handover on clinical outcomes has been addressed in several studies.^(^^5^^,^^6^^,^^19^^–^^22^^)^ For instance, a prospective study conducted in a tertiary ICU in Brazil showed that, in the absence of a handover protocol, diagnosis and treatment goals are either not communicated between or retained by intensivists immediately after handover in 50% to 60% of cases, demonstrating significant loss of information following transition between intensivist staffing.^(^^20^^)^ Therefore, handover processes must be improved in order to increase patient safety, reduce medical errors and prevent adverse events.^(^^21^^)^ Indeed, implementation of a handover protocol based on standardized oral and written handoff tools and communication training was associated with a 23% decrease in medical error and a 30% decrease in preventable adverse event rates.^(^^22^^)^

This study has limitations. Firstly, this study was conducted in a single ICU located in a private tertiary care hospital in Brazil. Therefore, findings may not be generalized to other intensive care units, since healthcare systems and patient characteristics may vary substantially between different cohorts. Secondly, propensity score matching was used in an effort to mitigate confounding and enhance the internal validity of the analysis. However, even though propensity score matching may have accounted for inherent differences in patient characteristics between groups, confounding cannot be ruled out entirely.^(^^16^^)^ Thirdly, given the exact duration of each handover was not documented, handover time was defined as the time periods from 6:30 am to 7:30 am and 6:30 pm to 7:30 pm. However, in the ICU in question, departing intensivists tended to begin to prepare for handover 30 minutes prior to handover time and the handover process usually lasted 30 minutes. Fourthly, the incidence of communication errors during handover and the impact of ICU admissions on handover process quality were not assessed. Communications problems, such as omissions and corruption of information, mostly due to distractions, have been pointed out as the leading causes of ineffective handover.^(^^10^^)^ Fifthly, data such as primary specialty of intensivists working at the ICU during the experimental period and their respective background as intensivists were not available. Experienced intensivists are expected to be more skilled in prioritization and handling of complex situations such as ICU admission during handover, without compromising patient safety. Finally, the impact of ICU admission during handover on ICU staff performance, patient and staff satisfaction and patient and family experience has not been investigated. These questions should be further evaluated.

## CONCLUSION

In this retrospective, propensity-matched, single-center cohort study, patient admission to the intensive care unit during handover was not associated with higher mortality rates or higher use of resources relative to admission at different times. Further large-scale, prospective multicenter studies are needed for deeper understanding of associations between admission during handover and outcomes.
