# Supplementary material for: Impact of intensive care unit admission during handover on mortality: propensity matched cohort study
Source: Einstein (Sao Paulo). 2021 Jun 10;19:eAO5748. doi: 10.31744/einstein_journal/2021AO5748 (PMC8225264; doi:10.31744/einstein_journal/2021AO5748)
Supplement: Translation

# Translation: Impacto na mortalidade da admissão de pacientes em unidade de terapia intensiva durante a passagem de plantão: estudo de coorte utilizando escore de propensão

**Authors:** Thais Dias Midega, Newton Carlos Viana Leite, Antonio Paulo Nassar, Roger Monteiro Alencar, Antonio Capone, Leonardo José Rolim Ferraz, Thiago Domingos Corrêa

**Affiliations:** 1 São PauloSP Brasil Hospital Israelita Albert Einstein, São Paulo, SP, Brasil.; 2 São PauloSP Brasil Hospital Municipal Dr. Moysés Deutsch; Hospital Israelita Albert Einstein, São Paulo, SP, Brasil.

**Keywords:** Transferência da responsabilidade pelo paciente, Segurança do paciente, Avaliação de resultados da assistência ao paciente, Unidades de terapia intensiva/estatística & dados numéricos, Comunicação, Readmissão do paciente, Alta do paciente, Mortalidade hospitalar, Recursos em saúde/estatística & dados numéricos

## Abstract

**Objetivo:**

Avaliar o impacto na mortalidade da admissão em unidade de terapia intensiva durante passagem de plantão médico.

**Métodos:**

Análise *post-hoc* de estudo original publicado previamente, com o objetivo de avaliar os impactos da readmissão em unidade de terapia intensiva nos desfechos clínicos. Este estudo de coorte retrospectivo, em centro único, com pareamento por escore de propensão, foi conduzido em uma unidade de terapia intensiva geral, aberta, com 41 leitos. Com base no tempo de internação na unidade de terapia intensiva, os pacientes foram categorizados em duas coortes: Grupo Passagem de Plantão (admissão entre 6h30 e 7h30 ou 18h30 e 19h30) ou Grupo Controle (internação entre 7h31 e 18h29 ou 19h31 e 6h29). Pacientes no Grupo Passagem de Plantão foram pareados com Grupo Controle na proporção de 1:2.

**Resultados:**

Entre 1° de junho de 2013 e 31 de maio de 2015, 6.650 pacientes adultos foram admitidos na unidade de terapia intensiva. Após a exclusão de participantes inelegíveis, 5.779 pacientes (389; 6,7% no Grupo de Admissão na Passagem de Plantão e 5.390; 93,3% no Grupo de Controle) foram elegíveis para pareamento por escore de propensão, dos quais 1.166 foram pareados com sucesso (389; 33,4% no Grupo Passagem de Plantão e 777; 66,6% no Grupo Controle). Após pareamento, admissão na unidade de terapia intensiva durante a passagem plantão não foi associada ao aumento da chance de óbito na unidade de terapia intensiva (RC: 1,40; IC95%: 0,92-2,11; p=0,113) ou no hospital (RC: 1,23; IC95%: 0,85-1,75; p=0,265).

**Conclusão:**

Internação em unidade de terapia intensiva durante passagem de plantão médico não impactou na mortalidade hospitalar.

## INTRODUÇÃO

A demanda global crescente por leitos de unidade de terapia intensiva (UTI) reflete o aumento da expectativa de vida e a prevalência crescente de enfermidades crônicas.^(^^1^^)^ Considerando a disponibilidade limitada de leitos de UTI, a melhoria das características organizacionais e operacionais dessa unidade é fundamental, para maior eficiência e melhores desfechos. No Brasil, a maioria da UTIs conta com intensivistas em tempo integral, isto é, 24 horas por dia, 7 dias por semana.^(^^2^^)^ Assim, a transferência frequente do cuidado entre profissionais da saúde é esperada.

A passagem do plantão consiste na transferência das informações do paciente, do planejamento terapêutico e das responsabilidades do profissional que está de saída para o profissional que chega.^(^^3^^)^ Associações entre passagem inadequada de plantão e desfechos clínicos já foram relatadas.^(^^4^^–^^6^^)^ Por exemplo, em um estudo realizado no departamento de emergência, a passagem inadequada de plantão teve efeito adversos sobre aproximadamente 5% dos pacientes, resultando em atraso da terapia.^(^^7^^)^ Uma revisão de queixas de imperícia no departamento de emergência revelou índice de 24% de falta de diagnóstico devido à passagem inadequada de plantão.^(^^8^^)^ Em outro estudo, a transferência intraoperatória de cuidados anestésicos entre anestesistas se traduziu em risco 43% mais alto de mortalidade hospitalar para pacientes submetidos à cirurgia cardíaca.^(^^9^^)^

O processo de passagem de plantão é particularmente desafiador na UTI, dada a complexidade dos pacientes graves e a propensão deles a alterações repentinas de condição clínica.^(^^10^^)^ O pacientes internados nessa unidade também tendem a ser instáveis e podem necessitar de manobras de ressuscitação, procedimentos invasivos e intervenções terapêuticas nas primeiras horas após a internação.^(^^11^^)^ Como consequência, a internação na UTI durante a passagem de plantão, quando os intensivistas se distanciam do cuidado direto do paciente, tem grande chance de aumentar a incidência de erros médicos e eventos adversos inesperados.^(^^12^^)^

## OBJETIVO

Avaliar o impacto na mortalidade hospitalar da internação de pacientes graves em unidade de terapia intensiva durante a passagem de plantão em um hospital terciário, e comparar o uso de recursos e os desfechos clínicos entre pacientes internados na unidade de terapia intensiva durante a passagem de plantão e pacientes internados em outros momentos.

## MÉTODOS

### Desenho experimental e local

Este estudo é uma análise *post-hoc* de dados extraídos de um estudo retrospectivo unicêntrico de coorte prévio que investigou os impactos da reinternação na UTI no uso de recursos e nos desfechos clínicos.^(^^13^^)^ O estudo original e esta análise *post-hoc* foram aprovados pelo Comitê de Ética do Hospital Israelita Albert Einstein (HIAE) e isentos de consentimento informado (CAAE: 54065716.3.0000.0071; parecer: 1.464.901).

### Local

Este estudo foi realizado em um hospital terciário, em São Paulo (SP). O hospital em questão contava com 662 leitos de internação, uma UTI geral aberta com 41 leitos e 91 leitos de unidade semi-intensiva.

### Pacientes

Pacientes consecutivos de idade superior a 18 anos internados na UTI entre 1° de junho de 2013 e 31 de maio de 2015 foram incluídos no estudo. Pacientes com dados fundamentais faltantes (idade, sexo, horário da internação na UTI, diagnóstico de internação na UTI, *Simplified Acute Physiology Score* – SAPS – III no momento da internação na UTI, duração da internação hospitalar e na UTI e estado vital no momento da alta) foram excluídos.

### Coleta de dados e variáveis estudadas

Os dados foram recuperados do sistema Epimed Monitor System^®^ (Epimed Solutions, Rio de Janeiro, RJ, Brasil). Esse sistema consiste em um formulário eletrônico estruturado de relatório de caso, no qual os dados do paciente são informados de forma prospectiva por gerenciadores de casos de UTI treinados.^(^^14^^)^ Foram coletadas as seguintes variáveis: características demográficas, comorbidades, localização antes da internação na UTI, horário da internação na UTI, SAPS III no momento da internação na UTI,^(^^15^^)^ diagnóstico de internação na UTI, necessidade de medidas invasivas de suporte (vasopressores, ventilação mecânica, ventilação mecânica não invasiva – VNI – ou terapia de substituição renal – TSR) na entrada e durante a permanência na UTI, destino após a alta, duração da internação hospitalar e na UTI, frequência de reinternação na UTI, mortalidade hospitalar, mortalidade no momento da alta da UTI e mortalidade com 90 dias.

### Definições

A passagem de plantão foi definida como a transferência dos cuidados do intensivista que estava de saída para o intensivista que chegava. O período de passagem de plantão estabelecido foi das 6h30 às 7h30 e das 18h30 às 19h30. Os pacientes foram alocados para uma das seguintes coortes, de acordo com o horário da internação na UTI: Grupo Passagem de Plantão (internação na UTI entre 6h30 e 7h30 ou entre 18h30 e 19h30) e Grupo Controle (internação entre 7h31 e 18h29 ou entre 19h31 e 6h29).

### Características da unidade de terapia intensiva e processo de passagem de plantão

A UTI contava com médicos no local 24 horas por dia, na proporção de um intensivista para cada dez leitos. Não havia redução de pessoal ou atividades nos turnos da noite ou aos finais de semana. Existiam reuniões clínicas multidisciplinares diárias envolvendo intensivistas, enfermeiros, fisioterapeutas especializados em fisioterapia respiratória, nutricionistas, psicólogos e farmacêuticos clínicos. As decisões referentes à internação na UTI eram de responsabilidade do intensivista de plantão, ao passo que a alta era um processo de tomada de decisão que envolvia o intensivista de plantão e o clínico responsável pelo paciente fora da UTI.^(^^13^^)^ Os intensivistas de plantão em dias de semana variaram, uma vez que os turnos eram de 12 horas.

A passagem de plantão foi realizada duas vezes por dia, às 7h e às 19h, quando terminava o turno do intensivista até então de plantão. O intensivista que estava de saída geralmente começava a preparar a passagem de plantão com 30 minutos de antecedência, e o processode passagem de plantão costuma durar 30 minutos. Não foram utilizados protocolos específicos e nem ferramentas padronizadas para direcionar a passagem de plantão durante o período experimental.

A passagem de plantão entre enfermeiros de UTI e fisioterapeutas respiratórios se deu no mesmo horário da passagem de plantão entre intensivistas.

### Análise estatística

As variáveis categóricas foram expressas na forma de frequências absolutas e relativas. As variáveis contínuas foram expressas na forma de medianas e intervalos interquatil (IIQ). A normalidade foi avaliada por meio do teste Kolmogorov-Smirnov. Os Grupos Passagem de Plantão e Controle foram comparados. As variáveis categóricas foram comparadas empregando-se o teste do *χ*^2^ ou o teste exato de Fisher, conforme o caso. As variáveis contínuas foram comparadas por meio do teste *t* para amostras independentes ou o teste U de Mann-Whitney para dados com distribuição anormal.

O pareamento baseado em escore de propensão foi empregado para consideração das diferenças entre as características dos pacientes, de forma a minimizar os efeitos de confusão. O escore de propensão de cada paciente do Grupo Passagem de Plantão foi estimado por meio de regressão logística baseada em 17 características relevantes (idade, sexo, SAPS III, motivo da internação na UTI, origem do encaminhamento, hipertensão, *diabetes mellitus*, câncer, insuficiência cardíaca congestiva, doença pulmonar obstrutiva crônica, doença renal crônica, doença renal crônica com necessidade de diálise de longo prazo, cirrose hepática e uso de vasopressores, uso de ventilação não invasiva, TSR ou ventilação mecânica durante a internação na UTI). Pacientes com dados faltantes foram excluídos. Uma coorte pareada, de acordo com o escore de propensão, foi construída com base em estimadores ponderados. O pareamento foi realizado empregando-se o algoritmo “vizinho mais próximo”, sem substituição, de tal forma que cada paciente do Grupo Passagem de Plantão foi pareado com dois pacientes do Grupo Controle. Uma calibração de largura de 0,10 do desvio-padrão do *logit* do escore de propensão foi empregada no desenvolvimento do pareamento.^(^^16^^,^^17^^)^

Os testes estatísticos foram bicaudais, e valores de p inferiores a 0,05 foram considerados significantes. Não foram realizados ajustes para multiplicidade nas análises. As análises estatísticas foram realizadas empregando-se o programa SPSS para Windows da IBM, versão 22.0. Os gráficos foram gerados empegando-se o programa GraphPad Prism para Windows, versão 6.00 (GraphPad Software, Califórnia, EUA).

## RESULTADOS

### Características da população estudada

Ao todo, 6.650 pacientes foram internados na UTI entre junho 2013 e maio 2015. Destes, 871 foram excluídos por falta de dados completos e/ou idade inferior a 18 anos. A amostra final incluiu 5.779 pacientes (389; 6,7% no Grupo Passagem de Plantão e 5.390; 93,3% no Grupo Controle). A mediana de idade dos pacientes nesta coorte foi de 67 (IIQ de 53 a 80) anos, com 56,5% de pacientes do sexo masculino. Dos 5.779 pacientes elegíveis para pareamento baseado em escore de propensão, 1.166 foram pareados com sucesso (389; 33,4% no Grupo Passagem de Plantão e 777; 66,6% no Grupo Controle) (Figura 1). A distribuição do horário de internação na UTI na população estudada (n=5.779 pacientes) pode ser visualizada no histograma da figura 2.

**Figura 1:** Fluxograma dos pacientes

**Figura 2:** Percentagem de internações na unidade de terapia intensiva por hora do dia

### Coorte antes do pareamento baseado em escore de propensão

As variáveis idade, sexo, SAPS III, motivo da internação na UTI, origem do encaminhamento, diagnóstico de internação, frequência de comorbidades e necessidade de ventilação mecânica, VNI, TSR ou vasopressores não diferiram entre os pacientes do Grupo Passagem de Plantão e os do Grupo Controle antes do pareamento baseado em escore de propensão (Tabela 1).

**Tabela 1:** Características dos participantes antes do pareamento baseado em escore de propensão

Características		Todos os pacientes 5.779 (100,0%)	Grupo Passagem de Plantão 389 (6,7%)	Grupo Controle 5.390 (93,3%)	Valor de p**
Idade, anos		67 (53-80)	67 (52-80)	66 (53-80)	0,483#
Sexo masculino		3.268 (56,5)	214 (55)	3.054 (56,7)	0,527&
SAPS III§		43 (33-55)	43 (32-54)	43 (33-55)	0,537‡
Motivo da internação na UTI					0,563&
	Médico	3.423 (59,2)	225 (57,8)	3.198 (59,3)	
	Cirúrgico	2.356 (40,8)	164 (42,2)	2.192 (40,7)	
Origem do encaminhamento					0,741&
	Sala cirúrgica/sala de procedimento	2.313 (40,0)	155 (39,8)	2.158 (40)	
	Departamento de emergência	2.217 (38,4)	148 (38,0)	2.069 (38,4)	
	Unidade de internação	557 (9,6)	35 (9,0)	522 (9,7)	
	Unidade semi-intensiva	377 (6,5)	24 (6,2)	353 (6,5)	
	Outra*	315 (5,5)	27 (6,9)	288 (5,3)	
Doença de base					
	Hipertensão	3.132 (54,2)	210 (54,0)	2.922 (54,2)	0,925&
	*Diabetes mellitus*	1.838 (31,8)	124 (31,9)	1.714 (31,8)	0,979&
	Câncer	1.270 (22,0)	97 (24,9)	1.173 (21,8)	0,145&
Insuficiência cardíaca congestiva		702 (12,2)	47 (12,1)	655 (12,2)	0,719&
	DPOC	519 (9,0)	36 (9,3)	483 (9,0)	0,854&
	Doença renal crônica com necessidade de diálise de longo prazo	470 (8,1)	25 (6,4)	445 (8,3)	0,202&
	Doença renal crônica	397 (6,9)	25 (6,4)	372 (6,9)	0,719&
	Cirrose hepática	279 (4,8)	21 (5,4)	258 (4,8)	0,588&
Diagnóstico de internação não cirúrgica					0,849&
	Sepse	1.657 (48,4)	113 (50,2)	1.544 (48,3)	
	Cardiovascular	508 (14,8)	38 (16,9)	470 (14,7)	
	Neurológico	384 (11,2)	22 (9,8)	362 (11,3)	
	Respiratório	326 (9,5)	22 (9,8)	304 (9,5)	
	Gastrintestinal	238 (6,7)	17 (7,6)	221 (6,9)	
	Metabólico	98 (2,9)	3 (1,3)	95 (3,0)	
	Trauma	94 (2,7)	4 (1,8)	90 (2,8)	
	Outros problemas médicos	69 (2,0)	3 (1,3)	66 (2,1)	
	Doenças renais	30 (0,9)	2 (0,9)	28 (0,9)	
	Causas hematológicas	19 (0,6)	1 (0,4)	18 (0,6)	
Diagnóstico de internação cirúrgica					0,748&
	Cardiovascular	643 (27,3)	42 (25,6)	601 (27,4)	
	Gastrintestinal	545 (23,1)	34 (20,7)	511 (23,3)	
	Ortopédico	394 (16,7)	26 (15,9)	368 (16,8)	
	Renal	286 (12,1)	25 (15,2)	261 (11,9)	
	Neurológico	235 (10,0)	15 (9,1)	220 (10,0)	
	Respiratório	203 (8,6)	19 (11,6)	184 (8,4)	
	Ginecológico	47 (2,0)	3 (1,8)	44 (2,0)	
	Trauma	3 (0,1)	0 (0,0)	3 (0,1)	
Suporte no momento da internação na UTI					
	Ventilação mecânica	953 (16,5)	57 (14,7)	896 (16,6)	0,312&
	Ventilação não invasiva	521 (9,0)	41 (10,5)	480 (8,9)	0,277&
	Vasopressores	852 (14,7)	48 (12,3)	804 (14,9)	0,166&
	TSR	24 (0,4)	0 (0,0)	24 (0,4)	0,187&
Destino no momento da alta da UTI					0,338&
	Unidade semi-intensiva	3.152 (54,5)	216 (55,5)	2.936 (54,5)	
	Unidade de internação	2.057 (35,6)	134 (34,4)	1.923 (35,7)	
	Outro/desconhecido*	237 (4,1)	11 (2,8)	226 (4,2)	

Resultados expressos por mediana (intervalo interquartil) ou n (%).

** os valores de p foram calculados da seguinte forma:

# teste *t*

& teste *χ*^2^ ou

‡ teste U de Mann-Whitney

§ escore SAPS III de 0 a 217, sendo escores mais altos indicativos de doença mais grave e maior risco de óbito

* outro hospital ou *home care*.

SAPS: *Simplified Acute Physiology Score*; UTI: unidade de terapia intensiva; DPOC: doença pulmonar obstrutiva crônica; TRS: terapia de substituição renal.

A mortalidade hospitalar foi de 14,1% (55/389 pacientes) e 11,7% (628/5.390 pacientes) no Grupo Passagem de Plantão e no Grupo Controle, respectivamente (razão de chance – RC: 1,25; intervalo de confiança de 95% – IC95%: 0,92-1,68; p=0,142). O uso de recursos, expresso como necessidade de vasopressores, ventilação mecânica, VNI ou TSR, não diferiu entre os grupos. A duração da internação hospitalar e a frequência de reinternação na UTI também foram semelhantes entre os grupos (Tabela 2).

**Tabela 2:** Desfechos antes do pareamento baseado em escore de propensão

Características		Todos os pacientes 5.779 (100,0%)	Grupo Passagem de Plantão 389 (6,7%)	Grupo Controle 5,390 (93,3%)	Valor de p*
Mortalidade na UTI		479 (8,3)	42 (10,8)	437 (8,1)	0,063†
Mortalidade com 90 dias		639 (11,1)	51 (13,1)	588 (10,9)	0,181†
Mortalidade hospitalar		683 (11,8)	55 (14,1)	628 (11,7)	0,142†
Suporte durante a internação na UTI					
	Vasopressores	1.586 (27,4)	98 (25,2)	1.488 (27,6)	0,303†
	Ventilação mecânica	1.401 (24,2)	88 (22,6)	1.313 (24,4)	0,440†
	Ventilação não invasiva	1.438 (24,9)	101 (26,0)	1.337 (24,8)	0,610†
	TSR	566 (9,8)	30 (7,7)	536 (9,9)	0,153†
Duração na internação na UTI, dias		2 (1-3)	2 (1-3)	2 (1-3)	0,117‡
Duração da internação hospitalar, dias		9 (5-20)	9 (9-18)	9 (5-20)	0,673‡
Reinternação na UTI		576 (10,0)	38 (9,8)	538 (10,0)	0,891†

Resultados expressos por mediana (intervalo interquartil) ou n (%).

* os valores de p foram calculados empregando-se:

† teste *χ*^2^ ou

‡ teste U de Mann-Whitney.

UTI: unidade de terapia intensiva; TRS: terapia de substituição renal.

### Coorte após o pareamento baseado em escore de propensão

A mediana de idade da coorte pareada com base no escore de propensão foi de 67 (IIQ de 53 a 80) anos, com 54,9% (640/1.166) de pacientes de sexo masculino e mediana do SAPS III de 43 (IIQ de 32 a 55) (Tabela 3). Os grupos estudados mostraram-se bem balanceados com relação às variáveis idade, sexo, SAPS III no momento da internação na UTI, motivo da internação na UTI, origem do encaminhamento, prevalência de comorbidades, diagnóstico de internação na UTI e necessidade de medidas de suporte no momento da internação na UTI (Tabela 3).

**Tabela 3:** Características dos participantes após o pareamento baseado em escore de propensão

Características		Todos os pacientes 1.166 (100,0%)	Grupo Passagem de Plantão 389 (33,4%)	Grupo Controle 777 (66,6%)	Valor de p**
Idade, anos		67 (53-80)	67 (52-80)	68 (53-80)	0,880#
Sexo masculino		640 (54,9)	214 (55)	426 (54,8)	0,952&
SAPS III§		43 (32-55)	43 (32-54)	43 (33-55)	0,621‡
Motivo da internação na UTI					0,750&
	Clínico	682 (58,5)	225 (57,8)	457 (58,8)	
	Cirúrgico	484 (41,5)	164 (42,2)	320 (41,2)	
Origem do encaminhamento					0,863&
	Sala cirúrgica/sala de procedimento	464 (39,8)	155 (39,8)	309 (39,8)	
	Departamento de emergência	454 (38,9)	148 (38,0)	306 (39,4)	
	Unidade de internação	91 (7,8)	35 (9,0)	56 (7,2)	
	Unidade semi-intensiva	76 (6,5)	24 (6,2)	52 (6,7)	
	Outra*	81 (6,9)	27 (6,9)	54 (6,9)	
Doença de base					
	Hipertensão	620 (53,2)	210 (54,0)	410 (52,8)	0,694&
	*Diabetes mellitus*	391 (33,5)	124 (31,9)	267 (34,4)	0,396&
	Câncer	305 (26,2)	97 (24,9)	208 (26,8)	0,502&
	Insuficiência cardíaca congestiva	152 (13,0)	47 (12,1)	105 (13,5)	0,494&
	DPOC	111 (9,5)	36 (9,3)	75 (9,7)	0,827&
	Doença renal crônica com necessidade de diálise de longo prazo	85 (7,3)	25 (6,4)	60 (7,7)	0,422&
	Doença renal crônica	76 (6,5)	25 (6,4)	51 (6,6)	0,929&
	Cirrose hepática	61 (5,2)	21 (5,4)	40 (5,1)	0,856&
Diagnóstico de internação não cirúrgico					0,579&
	Sepse	332 (48,7)	113 (50,2)	219 (47,9)	
	Cardiovascular	105 (15,4)	38 (16,9)	67 (14,7)	
	Neurológico	86 (12,6)	22 (9,8)	64 (14,0)	
	Respiratório	64 (9,4)	22 (9,8)	42 (9,2)	
	Gastrintestinal	44 (6,5)	17 (7,6)	27 (5,9)	
	Metabólico	17 (2,5)	3 (1,3)	14 (3,1)	
	Trauma	15 (2,2)	4 (1,8)	11 (2,4)	
	Outros problemas médicos	12 (1,8)	3 (1,3)	9 (2,0)	
	Doenças renais	3 (0,4)	2 (0,9)	1 (0,2)	
	Causas hematológicas	4 (0,6)	1 (0,4)	3 (0,7)	
Diagnóstico de internação operatório					0,876&
	Cardiovascular	116 (24,0)	42 (25,6)	74 (23,1)	
	Gastrintestinal	102 (21,1)	34 (20,7)	68 (21,3)	
	Ortopédico	76 (15,7)	26 (15,9)	50 (15,6)	
	Renal	70 (14,5)	25 (15,2)	45 (14,1)	
	Neurológico	54 (11,2)	15 (9,1)	39 (12,2)	
	Respiratório	52 (10,7)	19 (11,6)	33 (10,3)	
	Ginecológico	14 (2,9)	3 (1,8)	11 (3,4)	
Suporte no momento da internação na UTI					
	Ventilação mecânica	185 (15,9)	57 (14,7)	128 (16,5)	0,422&
	Ventilação não invasiva	113 (9,7)	41 (10,5)	72 (9,3)	0,488&
	Vasopressores	168 (14,4)	48 (12,3)	120 (15,4)	0,155&
	TSR	2 (0,2)	0 (0,0)	2 (0,3)	0,555&
Destino no momento da alta da UTI					0,723&
	Unidade semi-intensiva	645 (55,3)	216 (55,5)	429 (55,2)	
	Unidade de internação	407 (34,9)	134 (34,4)	273 (35,1)	
	Outro/desconhecido*	40 (3,4)	11 (2,8)	29 (3,7)	

Resultados expressos por mediana (intervalo interquartil) ou n (%).

** os valores de p foram calculados da seguinte forma:

# teste *t*

& teste *χ*^2^ ou

‡ teste U de Mann-Whitney

* outro hospital ou *home care*.

SAPS: *Simplified Acute Physiology* Score; UTI: unidade de terapia intensiva; DPOC: doença pulmonar obstrutiva crônica: TRS: terapia de substituição renal.

A mortalidade na UTI foi 10,8% (42/389 pacientes) e 8,0% (62/777 pacientes) no Grupo Passagem de Plantão e no Grupo Controle, respectivamente (RC: 1,40; IC95%: 0,92-2,11; p=0,113). A mortalidade hospitalar foi de 14,1% (55/389 pacientes) e 11,8% (92/777 pacientes) no Grupo Passagem de Plantão e no Grupo Controle, respectivamente (RC: 1,23; IC95%: 0,85-1,75; p=0,265). As variáveis necessidade de vasopressores, ventilação mecânica, VNI ou TSR, a duração da internação hospitalar e na UTI e a frequência de reinternação na UTI não diferiram entre os grupos (Tabela 4).

**Tabela 4:** Desfechos após o pareamento baseado em escore de propensão

Características		Todos os pacientes 1.166 (100,0%)	Grupo Passagem de Plantão 389 (33,4%)	Grupo Controle 777 (66,6%)	Valor de p*
Mortalidade na UTI		104 (8,9)	42 (10,8)	62 (8,0)	0,111†
Mortalidade com 90 dias		139 (11,9)	51 (13,1)	88 (11,3)	0,375†
Mortalidade hospitalar		147 (12,6)	55 (14,1)	92 (11,8)	0,265†
Suporte durante a internação na UTI					
	Vasopressores	302 (25,9)	98 (25,2)	204 (26,3)	0,696†
	Ventilação mecânica	270 (23,2)	88 (22,6)	182 (23,4)	0,760†
	Ventilação não invasiva	321 (27,5)	101 (26,0)	220 (28,3)	0,397†
	Terapia de substituição renal	94 (8,1)	30 (7,7)	64 (8,2)	0,756†
Duração na internação na UTI, dias		2 (1-4)	2 (1-3)	2 (1-4)	0,258‡
Duração na internação hospitalar, dias		10 (5-20)	9 (5-18)	10 (5-20)	0,689‡
Reinternação na UTI		120 (10,3)	38 (9,8)	82 (10,6)	0,678†

Resultados expressos por mediana (intervalo interquartil) ou n (%).

* os valores de p foram calculados empregando-se

† teste *χ*^2^ ou

‡ teste U de Mann-Whitney.

UTI: unidade de terapia intensiva.

## DISCUSSÃO

O principal achado deste estudo retrospectivo unicêntrico de coorte pareada com base em escore de propensão foi que a internação na UTI durante a passagem de plantão não afetou o uso de recursos e nem os desfechos dos pacientes.

A ideia de que a internação durante a passagem de plantão possa influenciar no cuidado do paciente de urgência foi abordada recentemente em estudo retrospectivo unicêntrico de coorte com pacientes em sepse que chegaram à emergência durante a passagem de plantão da enfermagem.^(^^3^^)^ O estudo em questão não revelou diferenças significantes no tempo até a administração de antibióticos, no tempo para obtenção de dosagem de lactato sérico e cultura sanguínea e nem na mortalidade hospitalar entre os pacientes que chegaram durante a passagem de plantão e os demais.^(^^3^^)^

Até onde se sabe o impacto da internação na unidade de terapia intensiva durante a passagem de plantão sobre os desfechos clínicos ainda não foi estudado. Entretanto, há evidências de que a transferência entre médicos do cuidado do paciente cirúrgico tenha relação com piores desfechos. O impacto da transferência intraoperatória total do cuidado anestésico entre anestesiologistas foi comparado com a situação oposta em um estudo populacional retrospectivo de coorte com 313.066 pacientes que passaram por cirurgias grandes no Canadá.^(^^18^^)^ No estudo em questão, o risco de eventos adversos pós-operatórios foi mais elevado entre os pacientes que passaram pela transição do cuidado médico durante a cirurgia.^(^^18^^)^ Em outro estudo retrospectivo unicêntrico, a transferência do cuidado anestésico durante a cirurgia esteve associada a um risco 43% mais alto de mortalidade hospitalar.^(^^9^^)^ Analogamente, pacientes que passam por transferência de cuidados anestésicos durante a cirurgia têm desfechos piores do que os demais, principalmente devido à perda de informação.^(^^18^^)^

O impacto dos erros de comunicação durante a passagem de plantão sobre os desfechos clínicos foi abordado em diversos estudos.^(^^5^^,^^6^^,^^19^^–^^22^^)^ Por exemplo, estudo prospectivo realizado em uma UTI terciária no Brasil mostrou que, na ausência de um protocolo de passagem de plantão, o diagnóstico e os objetivos do tratamento deixam de ser comunicados ou recordados pelos intensivistas logo após a passagem de plantão em 50% a 60% dos casos, demonstrando perda significativa de informação na transição entre intensivistas.^(^^20^^)^ Portanto, o processo de passagem de plantão precisa ser melhorado, a fim de aumentar a segurança do paciente, diminuir a ocorrência de erros médicos e prevenir eventos adversos.^(^^21^^)^ De fato, a implementação de um protocolo de passagem de plantão baseado em ferramentas orais e escritas padronizadas de transferência do cuidado levou à queda de 23% na incidência de erros médicos e de 30% nos índices de eventos adversos passíveis de prevenção.^(^^22^^)^

Este estudo teve limitações. Em primeiro lugar, o estudo foi realizado em uma única UTI de um hospital terciário brasileiro. Assim, os achados podem não ser generalizados para outras UTIs, uma vez que os sistemas de saúde e as características dos pacientes variam consideravelmente entre coortes distintas. Em segundo lugar, o pareamento baseado em escore de propensão foi empregado numa tentativa de mitigar os efeitos de confusão e aumentar a validade da análise. Entretanto, embora o pareamento baseado em escore de propensão tenha levado em conta as diferenças entre as características dos pacientes entre os grupos, o efeito de confusão não pode ser totalmente descartado.^(^^16^^)^ Em terceiro lugar, uma vez que a duração exata de cada passagem de plantão não foi documentada, o período de passagem de plantão estabelecido foi das 6h30 às 7h30 e das 18h30 às 19h30 da noite. Entretanto, na UTI estudada, os intensivistas que estavam de saída começavam a preparar a passagem de plantão com 30 minutos de antecedência, e o processo de passagem de plantão costumava durar 30 minutos. Em quarto lugar, a incidência de erros de comunicação durante a passagem de plantão e o impacto das internações na UTI sobre a qualidade do processo de passagem de plantão não foram avaliados. Problemas de comunicação, como omissões e corrupção de informações, sobretudo devido a distrações, foram apontados como a principal causa de passagem inadequada de plantão.^(^^10^^)^ Em quinto lugar, dados como a especialidade médica dos intensivistas de plantão durante o período experimental e a experiência deles no cuidado intensivo não estavam disponíveis. Espera-seque intensivistas experientes sejam mais capazes de estabelecer prioridades e lidar com situações complexas, como a internação na UTI durante passagem de plantão, sem comprometer a segurança do paciente. Finalmente, o impacto da internação na UTI durante a passagem de plantão sobre o desempenho da equipe, a satisfação da equipe e do paciente e a experiência do paciente e de sua família não foram investigados. Essas questões são dignas de serem abordadas mais a fundo.

## CONCLUSÃO

Neste estudo retrospectivo unicêntrico de coorte com pareamento baseado em escore de propensão, a internação do paciente na unidade de terapia intensiva durante a passagem de plantão não se mostrou associada a maior mortalidade e nem a maior uso de recursos em relação à internação em outros momentos. Estudos prospectivos multicêntricos de larga escala se fazem necessários para melhor compreensão das associações entre internação durante a passagem de plantão e desfechos clínicos.
